# Compressed Sensing for Biomedical Photoacoustic Imaging: A Review

**DOI:** 10.3390/s24092670

**Published:** 2024-04-23

**Authors:** Yuanmao Wang, Yang Chen, Yongjian Zhao, Siyu Liu

**Affiliations:** 1 School of Physics, Nanjing University of Science and Technology, Nanjing 210094, China; 2 Department of Electronic Engineering, The Chinese University of Hong Kong, Hong Kong 999077, China; 3 Southwest Institute of Technical Physics, Chengdu 610041, China

**Keywords:** biomedical imaging, photoacoustic technique, compressed sensing

## Abstract

Photoacoustic imaging (PAI) is a rapidly developing emerging non-invasive biomedical imaging technique that combines the strong contrast from optical absorption imaging and the high resolution from acoustic imaging. Abnormal biological tissues (such as tumors and inflammation) generate different levels of thermal expansion after absorbing optical energy, producing distinct acoustic signals from normal tissues. This technique can detect small tissue lesions in biological tissues and has demonstrated significant potential for applications in tumor research, melanoma detection, and cardiovascular disease diagnosis. During the process of collecting photoacoustic signals in a PAI system, various factors can influence the signals, such as absorption, scattering, and attenuation in biological tissues. A single ultrasound transducer cannot provide sufficient information to reconstruct high-precision photoacoustic images. To obtain more accurate and clear image reconstruction results, PAI systems typically use a large number of ultrasound transducers to collect multi-channel signals from different angles and positions, thereby acquiring more information about the photoacoustic signals. Therefore, to reconstruct high-quality photoacoustic images, PAI systems require a significant number of measurement signals, which can result in substantial hardware and time costs. Compressed sensing is an algorithm that breaks through the Nyquist sampling theorem and can reconstruct the original signal with a small number of measurement signals. PAI based on compressed sensing has made breakthroughs over the past decade, enabling the reconstruction of low artifacts and high-quality images with a small number of photoacoustic measurement signals, improving time efficiency, and reducing hardware costs. This article provides a detailed introduction to PAI based on compressed sensing, such as the physical transmission model-based compressed sensing method, two-stage reconstruction-based compressed sensing method, and single-pixel camera-based compressed sensing method. Challenges and future perspectives of compressed sensing-based PAI are also discussed.

## 1. Introduction

Humans have entered the digital age, where signal processing has shifted from analog to digital domain, and many commonly used technologies have also transitioned from analog devices to digital devices [1,2,3,4]. This transformation is increasingly significant due to the superior controllability, flexibility, low cost, and ease of popularization of digital signals. The success of digital signals has driven the development of digital information in sampling systems, with the Nyquist sampling theorem playing a crucial role in this process [5,6]. The theorem states: “In the process of converting analog signals to digital signals, when the sampling frequency is greater than twice the highest frequency in the signal, the sampled digital signal can completely retain the information from the original signal [7,8]”. However, the Nyquist sampling theorem often leads to redundant sampling [9,10], resulting in substantial costs to meet the sampling rate requirements in certain applications. Despite the ongoing advancements in various aspects of computers, there are still many challenges in data acquisition and processing [11].

To address the storage and transmission challenges encountered when dealing with multi-dimensional massive data, compression techniques are commonly employed. This involves refining the original digital signal representation to reduce the demands on storage space and transmission bandwidth. Subsequently, various compression technologies have emerged, including lossless compression and lossy compression [12,13,14]. The emergence of Compressed Sensing (CS) has provided a new direction for compression technology. CS is capable of retaining essential data, discarding significant amounts of redundant data, and reconstructing the original data with redundancy using a small amount of essential data through known mapping relationships [15,16,17,18]. CS has been widely applied in the field of signal processing since its introduction [19,20,21,22,23,24].

PAI, as a non-invasive medical imaging method, combines the advantages of high-contrast optical imaging and deep penetration of ultrasound imaging [25,26,27]. In recent years, the introduction of CS has injected new momentum into the development of PAI. Typically, CS is applied in PAI to address the challenges of high-dimensional data acquisition and processing in signal processing. From a signal processing perspective, introducing CS into PAI provides advantages in photoacoustic signal processing [28]. Firstly, PAI systems involve a significant amount of signal acquisition, whereas traditional PAI requires collecting a large amount of complete signals for image reconstruction. This results in high signal dimensions and quantities, posing challenges for signal processing and storage [29]. However, CS, utilizing the sparsity or structural information of signals, can capture key information with fewer sampling points, effectively reducing signal dimensions and sampling amounts [30]. Secondly, CS exhibits strong adaptability and robustness in signal reconstruction [31]. Even in the presence of noise or incomplete sampling during the acquisition process, CS can accurately reconstruct the original signal. In PAI, signals may be subject to various interferences, and the collected signals may not be complete. CS can leverage its advantages in the reconstruction stage to address this issue, improving reconstruction quality and ensuring the quality of photoacoustic images. Additionally, CS offers the advantages of rapid reconstruction [32]. Compared to the complex computational processes of traditional methods, CS can quickly reconstruct signals through simple mathematical models and iterative algorithms, providing significant advantages in real-time imaging and rapid diagnosis [33].

CS-based PAI fully utilizes the advantages of sparse representation, robust reconstruction, and fast processing provided by CS. It achieves efficient acquisition, processing, and reconstruction of photoacoustic signals, bringing higher efficiency, more cost-effectiveness, and real-time imaging capabilities to PAI systems [34]. It has promoted the application and development of PAI in medical diagnosis and life science research. Notably, the introduction of CS into PAI also solves the problem of limited viewing angles. This is a common and important challenge in PAI, where sensors are unable to fully cover the target area, resulting in missing information and degraded image quality during image reconstruction [35,36]. It is necessary to combine the signal sparsity hypothesis, reasonable design of the sampling mode, sparse representation, reconstruction algorithm, and prior information so as to realize efficient acquisition and reconstruction of limited viewing angle photoacoustic signals and improve imaging quality and accuracy. This method can effectively deal with the challenge of limited perspective in the practical application of PAI, expanding the application prospect of PAI in the field of medical imaging.

In the following, we will review recent CS-based PAI research.

## 2. Biomedical Photoacoustic Technique

### 2.1. Photoacoustic Effect

PAI is a newly emerging hybrid imaging technique based on photoacoustic effect [37], integrating the advantages of both acoustic and optical imaging [38,39]. Since A.G. Bell discovered the photoacoustic effect in 1880, there was a long period of stagnation until the breakthrough advancements in laser technology in the 1960s enabled the widespread adoption of photoacoustic technology in industrial and scientific fields [40]. Currently, PAI has made remarkable progress internationally, enabling imaging of partial tissues in animals and humans (such as capillaries, melanin, and tumors) [41], as well as allowing imaging of the brain and limbs of living mice [27,42,43].

From the above introduction, it can be seen that PAI possesses both optical and acoustic characteristics. Optically, PAI utilizes the optical absorption parameters of biological tissue as an imaging parameter, which is closely related to the composition of biological tissue [44,45]. Therefore, PAI can acquire information about tissue composition, further reflecting the functional information of tissues. The scattering of ultrasound waves in tissues is two to three orders of magnitude weaker than optical scattering. Therefore, when the propagation distance of light in tissues exceeds the average mean free path, PAI can achieve higher acoustic resolution. Compared with traditional optical imaging techniques, PAI can achieve high-resolution imaging of optical absorption in deep tissues. In addition, in biological tissues, with nanosecond pulsed laser irradiation, approximately 800 Pa of initial pressure increase is generated for every 1 mK temperature rise [46,47,48,49,50]. 

When a substance is irradiated by a laser, the irradiated area and its surrounding environment absorb energy and convert it into heat, causing changes in stress or pressure, thereby exciting and propagating acoustic waves [25,26,51,52]. The intensity and phase of the photoacoustic signals are not only related to the light source but also to the spatial distribution of the optical absorption coefficient of the irradiated substance and its optical, thermal, and elastic properties. By detecting the photoacoustic signals generated by the photoacoustic effect, PAI can invert the optical properties of materials within the imaging area and reconstruct images inside the irradiated region [53,54].

Many valuable works have described the principles of PAI before [26,45,55,56,57]. In PAI, the initial photoacoustic pressure can be expressed as

(1) $$p_{0}=\Gamma_{0}\eta_{th}\mu_{a}F$$

where $\Gamma_{0}$ is the Gruneisen parameter of the tissue, $\eta_{th}$ is the energy conversion efficiency of light to heat, $\mu_{a}$ is the optical absorption coefficient, and F is the local optical influence. It can be observed that the magnitude of the initial photoacoustic pressure excited by the laser is directly proportional to the optical absorption coefficient, which in turn indicates the distribution within biological tissues. Therefore, as long as the initial photoacoustic pressure can be inverted through the received photoacoustic signals, the imaging objective can be achieved.

Without considering the influence of viscoelastic factors, the propagation of photoacoustic signals in the medium of thermal expansion stage can be expressed by the following photoacoustic equation:

(2) $$(\nabla^{2}-\frac{1}{v_{s}^{2}}\frac{\partial^{2}}{\partial t^{2}})p(r,t)=-\frac{\beta }{\kappa v_{s}^{2}}\frac{\partial^{2}T(r,t)}{\partial t^{2}}$$

where p(r,t) is the photoacoustic pressure at position r and time t, T is the temperature rise, κ is the isothermal compression coefficient, β is the thermal coefficient of volume expansion, and $v_{s}$ is the speed of sound. In PAI, the short laser pulse duration should meet two constraints: the thermal diffusion time and stress relaxation time. The duration of the laser pulse should be much shorter than these two times, and its heat equation is expressed as

(3) $$pC_{V}\frac{\partial T(r,t)}{\partial t}=H(r,t)$$

If the laser pulse duration meets the above conditions. H represents the heating function, $C_{v}$ is the specific heat capacity of the equal body, and H(r,t) represents the heat energy converted per unit volume and per unit time. Substituting Equation (3) into Equation (2) yields

(4) $$(\nabla^{2}-\frac{1}{v_{s}^{2}}\frac{\partial^{2}}{\partial t^{2}})p(r,t)=-\frac{\beta }{C_{p}}\frac{\partial H(r,t)}{\partial t}$$

As can be seen from the above formula, since the source term is related to the first time derivative of H, time-invariant heating will not produce pressure waves, and photoacoustic signals will only be generated with time-varying heating. $C_{p}$ is the isobaric-specific heat capacity. This equation can be solved using the Green function [55]:

(5) $$p(r,t)=\frac{1}{4\pi v_{s}^{2}}\frac{\partial }{\partial t}\left[\frac{1}{v_{s}t}\int dr^{\prime }p_{0}(r^{\prime })\delta (t-\frac{\left|r-r^{\prime }\right|}{v_{s}})\right]$$

In the above formula, $p_{0}(r^{\prime })$ is the initial pressure at position $r^{\prime }$. Both sides of Equation (5) can be Fourier-transformed at the same time to push the photoacoustic equation in the time domain to the frequency domain.

### 2.2. Biomedical Photoacoustic Imaging

**Figure 1:**
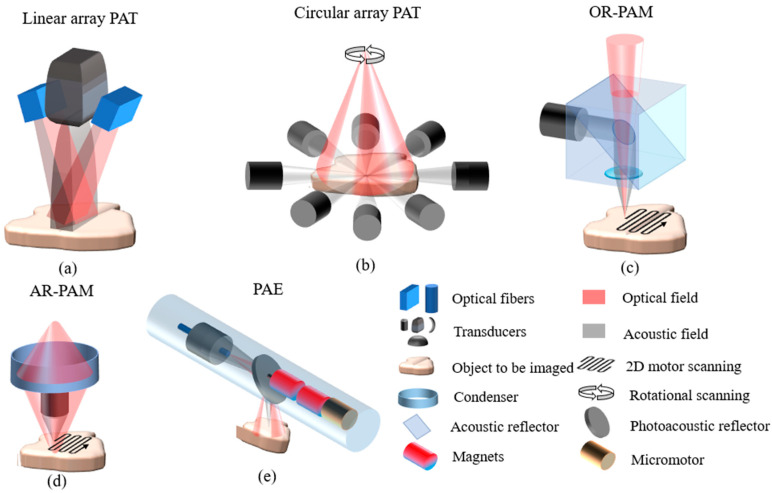
Schematic diagram of photoacoustic imaging mode. (**a**) Linear array PAT. (**b**) Circular array PAT. (**c**) OR-PAM. (**d**) AR-PAM. (**e**) PAE.

PAI can be realized through several configurations, including photoacoustic tomography (PAT), photoacoustic microscopy (PAM), and photoacoustic endoscopy imaging (PAE) [58]; schematic diagrams of various photoacoustic imaging modalities are shown in Figure 1.

PAT uses a non-focusing large-diameter pulsed laser beam to achieve full-field illumination of the tissue surface [44,59,60]. The absorption of incident light energy by biological tissues leads to thermal expansion, which results in the rapid generation of broadband ultrasound waves. The ultrasound waves propagate to the tissue surface, and the photoacoustic signals are acquired by a mechanically scanned unfocused ultrasound transducer or transducer array. By solving the inverse problem of photoacoustic propagation, the spatial distribution of the relative optical absorption coefficient of the imaged region is reversely deduced from the detected photoacoustic signals, and the photoacoustic images of the imaging region are reconstructed accordingly. The image reconstruction of PAT relies on specific image reconstruction algorithms; commonly used reconstruction algorithms include the back-projection method [61], Radon transform [62], and Fourier transform algorithm [63]. PAT is mainly divided into two imaging modes: linear array PAT (Figure 1a) and circular array PAT (Figure 1b). 

In the PAM mode, the laser is focused on the surface of the sample being imaged, while a point-focused ultrasound transducer is used to detect the photoacoustic signals generated at the laser focus. The optical focusing lens and the focused ultrasound transducer are usually confocal to maximize detection sensitivity. The arrival time of the photoacoustic signal corresponds to the distance between the sound source and the ultrasound transducer [64]. PAM obtains images through point-by-point scanning. Based on the relative sizes of the optical beam focus spot and the acoustic beam focus spot in the imaging system, PAM can be divided into optical resolution photoacoustic microscopy (OR-PAM) (Figure 1c) and acoustic resolution photoacoustic microscopy (AR-PAM) (Figure 1d). Currently, OR-PAM has more applications than AR-PAM [65]. AR-PAM typically uses a single mechanically translated or rotated focused sensor to receive the photoacoustic signals [66]. The optical focusing of OR-PAM is tighter than the acoustic focusing, the optical focus is smaller than the acoustic detection focus, and its lateral resolution depends on the size of the optical focus, achieving sub-cellular or cellular scales ranging from nanometers to micrometers. However, due to the strong scattering effect of biological tissues on lasers, the imaging depth of in vivo OR-PAM is limited to 1–2 mm [67]. 

PAE is a special form of PAM that has been rapidly developed in recent years for examining the internal organs of the human body (such as the esophagus and colon) [68,69]. Its basic imaging principle is the same as that of PAM, but the difference is that PAE miniaturizes the imaging system and adopts a special scanning mechanism (Figure 1e) to meet the needs of in-depth examination inside the human body. The main goal of PAM and PAE is to achieve micron-scale resolution at millimeter-scale imaging depths.

In PAI, the most widely studied and applied modalities are PAT and PAM [47,70,71]. There are some differences between PAT and PAM. The imaging scales of the two modalities are different; PAT is suitable for acquiring three-dimensional holographic imaging of entire tissues or organs, while PAM is mainly used for microscopic-level imaging of microstructures. The resolution of the two modalities is different; PAT has a relatively lower spatial resolution, generally ranging from hundreds of micrometers to several millimeters, while PAM has a higher spatial resolution, typically ranging from tens to hundreds of micrometers. The scope of application of the two modalities is also different; PAT is widely used in medical imaging of tumors, brain function research, etc., while PAM is mainly used for imaging microstructures such as cells, capillaries, and neurons.

### 2.3. Biomedical Application of Photoacoustic Technique 

PAI has rapidly developed in recent years and has successfully achieved high-resolution image reconstruction of different tissues, thus being highly anticipated in clinical medicine [25,26,27,58,71]. PAI achieves tissue imaging by avoiding ionizing radiation that can harm the human body and the need for contrast agents, and it can provide real-time display. PAI plays an important role in many clinical applications. For example, it involves non-invasive imaging of human tissues, including the breast, sentinel lymph nodes, skin, thyroid, eyes, prostate (via rectum), ovaries (via vagina) [72], minimally invasive endoscopic imaging of the gastrointestinal tract, bladder, circulating tumor cells (in vivo flow cytometry), intraoperative tumor margin imaging, and lymph node metastasis imaging. Overall, in preclinical research, PAI is mainly applied in areas such as brain injury, disease prediction, tumor metastasis, and cancer diagnosis. Additionally, PAI technology has shown promising results in studying diseases related to vascular structure and function, such as stroke, epilepsy, and traumatic brain injury [73]. Significant progress has been made in non-invasive studies of organs or tissue in small animals such as mice, rabbits, and dogs, laying the foundation for future groundbreaking medical imaging technologies [27]. PAI has shown promising results in animal experiments, but what is even more encouraging is its significant imaging effectiveness on human tissue and organs. Furthermore, these techniques have undergone thorough application analysis and stage-wise validation. These studies are of great significance for understanding the development of human diseases and developing new drugs and treatment methods.

PAI has achieved success as an emerging biomedical imaging technology but also faces various challenges [74]. Firstly, the high sampling rate of PAI systems may lead to data redundancy and high hardware costs. Secondly, limited viewing angles and sparse transducer arrays can decrease imaging quality. Finally, the computation speed may be reduced when imaging algorithms involve large matrix operations. Additionally, improving imaging accuracy and precision has always been a goal of PAI. Therefore, some researchers have attempted to introduce compressed sensing to address the common issues in PAI, which will be detailed in the fourth section of this paper.

## 3. Compressed Sensing 

CS is a signal processing concept proposed by David Donoho and others [15,16,19,75], which is a technique for finding sparse solutions to underdetermined linear systems. CS is a signal-processing technique that can achieve signals with much lower sampling rates than those required by traditional sampling theory. CS takes advantage of the sparsity of signals, and through sparse representation and compression during the sampling process, it can obtain sampled signals at much lower rates than the Nyquist sampling rate. This means that a large number of traditional sampling points can be skipped in the signal recovery process, thereby reducing sampling and storage costs.

In terms of signal storage and transmission, in order to reduce the cost of storage and transmission, we often use compression methods that represent signals with fewer bits, discarding a large amount of non-important data. This process of high-speed sampling, followed by compression, wastes a significant amount of sampling resources. CS addresses this issue by using alternative transform spaces to describe signals (such as Fourier transform) and establishing a new theoretical framework for signal description and processing [76]. By ensuring no loss of information, signals can be sampled at rates far below those required by the Nyquist sampling theorem while still being able to fully recover the signal. With this problem solved, CS greatly reduces the sampling frequency and the cost of signal storage and transmission, significantly lowers the signal processing time and computational costs, and will lead signal processing into a new revolutionary era [77,78].

In terms of signal reconstruction, according to the Nyquist–Shannon sampling theorem, in order to fully recover a signal, it is necessary to sample at least twice the bandwidth of the signal. CS theory is different from traditional sampling theorems. As long as the signal is compressible or sparse in some transform domain, it is possible to use an observation matrix that is unrelated to the transformation basis to project the high-dimensional signal obtained from the transformation onto a low-dimensional space. Then, by solving an optimization problem, the original signals can be reconstructed with high probability from these few projections. It can be proven that such projections contain sufficient information for reconstructing the signal [18,22,79]. In this theoretical framework, the sampling rate is not determined by the signal bandwidth but by the structure and content of information in the signal.

However, in many practical applications, the effective information of a signal typically only occupies a small portion of the entire signal space; such signals exhibit sparsity. The basic principle of CS mainly consists of three parts: sparse representation, compressed sensing, and signal reconstruction. The three parts have also been the focus of research by CS researchers in recent years. 

### 3.1. Sparse Representation

All signals in nature can be divided into two types: sparse signals and non-sparse signals [80]. If a signal itself is already sparse, there is no need to represent the signal sparsely when applying CS. For non-sparse signals, in order to express these signals more concisely, the signal can typically be transformed into a new basis or framework. When the number of non-zero coefficients is much smaller than the number of terms in the original signal, these few non-zero coefficients can be considered as the sparsity representation of the original signal. In scenarios where storage space or transmission bandwidth is limited, only storing or transmitting non-zero coefficients in some basis or framework, rather than the entire original redundant signal, can be more efficient. Therefore, this sparsity representation has significant practical implications [22,30,81]. In the theoretical framework of CS, a sparse signal model can ensure high compression ratios. As long as it is known in advance that the target signal has a sparse representation in a known basis or framework, the original signal can be reconstructed without distortion. Popular methods for sparse signal representation are mostly based on sparse transforms, such as Fourier transform [79], wavelet transform [82], and discrete cosine transform [83], all of which exhibit a certain degree of sparsity. The sparse decomposition in a redundant dictionary is a hot research topic in sparse representation, and the construction of sparse dictionaries focuses on overcomplete dictionaries. The basic criterion for designing or learning a redundant dictionary is to match the inherent characteristics of the signal as closely as possible during the dictionary construction process. Choosing an appropriate sparse dictionary can ensure that the signal representation coefficients are sparse enough, thereby reducing the number of compressed sensing measurements related to non-zero coefficients and reconstructing the signal with high probability. Both sparse transform bases and sparse dictionaries satisfy the mathematical model of sparse representation in CS [16]:

(6) X=ΨS

where X is a one-dimensional non-sparse signal; Ψ is a sparse transform basis or a sparse dictionary; S is a sparse coefficient; and there are only non-zero values in S, which are much smaller than the number of dimensions of the X signal.

### 3.2. Compression Measurement

**Figure 2:**
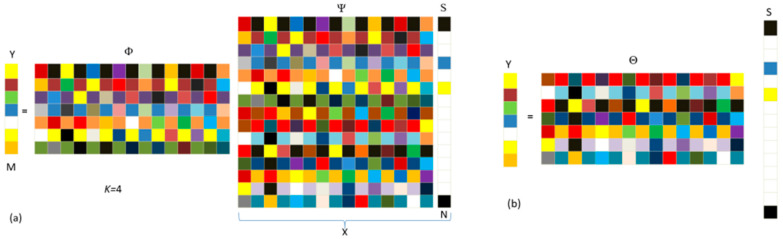
CS mathematical model. (**a**) CS mathematical model with sparse transformation matrix and measurement matrix (**b**) CS mathematical model with a sensing matrix.

To ensure the accurate reconstruction of the original signal, constructing a measurement matrix is crucial [10,15,84]. The role of the measurement matrix is to project the high-dimensional signal onto a low-dimensional space, reducing the signal’s dimensions. It is important to note that the constructed measurement matrix should be unrelated to the sparse transform matrix, and the measurement matrix should satisfy the Restricted Isometry Property (RIP) [85]. Currently, popular and stable performance measurement matrices include Gaussian random matrices, Hadamard matrices, Bernoulli matrices, sparse random matrices, and Toeplitz matrices [86]. The compression measurement step is a critical part of reducing computational complexity in CS, and it is also an important manifestation process of the CS mathematical model, as shown in Figure 2.

Figure 2 depicts a simplified schematic diagram of the CS compression measurement process involving two different mathematical models [15]:

(7) Y=ΦX=ΦΨS=ΘS

where Y is the M×1 vector, which is the compression measurement value; Φ is the M×N matrix, is the measurement matrix; Ψ is an N×N matrix, a sparse transformation matrix; Θ is the M×N matrix, which is the sensing matrix; and S is the N×1 vector and the sparse coefficient derived from the sparse transformation matrix. The K value in Figure 2a represents the number of non-zero values in the vector. Note: N>M≫K. In this way, a high-dimensional signal is projected into a low-dimensional signal, and the sensing matrix connects the information of the two, making reconstruction possible.

### 3.3. Signal Reconstruction

The signal reconstruction in CS is the process of recovering the original high-dimensional signal from the compressed low-dimensional signal through a sensing matrix and designed optimization algorithms. The entire process is essentially solving an underdetermined system of equations. There are three common types of reconstruction methods: convex optimization algorithms [87], greedy algorithms [88], and Bayesian algorithms [89]. By using these optimization algorithms, the low-dimensional signal Y can be rapidly reconstructed to the original signal X.

Convex optimization methods solve based on minimizing the $L_{1}$ norm, which leads to better reconstruction results compared to other algorithms. However, due to its high computational complexity and time-consuming nature, it is not widely used in large-scale signal processing. Nevertheless, in recent years, some convex optimization methods have achieved faster reconstruction speeds in reconstructing sparse signals. For example, the Alternating Direction Method of Multipliers treats convex optimization problems differently from other algorithms by not only viewing them as general minimization problems but also considering their separable structure. By solving each variable separately in solving convex optimization problems, the algorithm’s speed is significantly improved [90,91].

Compared to convex optimization algorithms based on minimizing the $L_{1}$ norm, greedy pursuit algorithms have a fast computation speed. Although the accuracy is slightly lower, it can still meet the general requirements of practical applications [88]. Therefore, greedy pursuit algorithms based on minimizing the $L_{0}$ norm are very practical and widely used. These algorithms solve the $L_{0}$ norm minimization problem, and improved versions of these algorithms allow for certain errors during the reconstruction process. In addition, the Iterative Thresholding Algorithm has also been widely applied. This type of algorithm is relatively easy to implement, with moderate computational complexity, and finds applications in both greedy and convex optimization algorithms. However, the Iterative Thresholding Algorithm is sensitive to the choice of initial values and thresholds and cannot guarantee that the solution obtained is sparse. In greedy algorithms, the most representative ones are Matching Pursuit (MP) and Orthogonal Matching Pursuit (OMP). MP is the most primitive signal sparse reconstruction algorithm, which is an iterative algorithm that seeks the sparse representation of a signal through step-by-step approximation. It defines a normalized basic module representing the signal space as the measurement matrix. These normalized vectors are called atoms. If the atoms of the measurement matrix span the entire signal space, then the measurement matrix is complete. If there is a linear dependence between atoms, the dictionary is redundant. In most applications of MP, the measurement matrix is both complete and redundant [92]. The OMP algorithm follows the same method as the MP algorithm in selecting atoms. It selects a column vector from the measurement matrix that is closest to the original signal or residual. The most significant difference from the MP algorithm is that when selecting the column vector closest to the original signal, the OMP algorithm performs an orthogonalization operation on it. This is to ensure that the OMP algorithm does not repeatedly select a column vector that has already been chosen from the measurement matrix [93,94,95].

The CS Bayesian algorithm is a signal-reconstruction method based on Bayesian statistical theory, which combines the ideas of CS and Bayesian inference. This algorithm models the signal using the Bayesian framework and achieves sparse reconstruction through maximum a posteriori probability estimation [89,96,97]. In the CS Bayesian algorithm, it is assumed that the signal to be reconstructed is a random variable from a prior distribution. By updating the prior distribution of the signal with compressed measurements, the posterior distribution of the signal is obtained. Ultimately, by maximizing the posterior probability, the signal with the highest posterior probability is chosen as the reconstruction result. The algorithm first establishes the prior distribution of the signal and models the relationship between the prior distribution and the compressed measurements as a conditional probability distribution. Then, according to Bayes’ theorem, the observation data are used to update the posterior distribution of the signal. Finally, by maximizing the posterior probability, a sparse representation or an approximate solution is reconstructed. The advantage of the CS Bayesian algorithm is that it can utilize prior information to model the signal more accurately, thereby improving the accuracy and stability of the reconstruction. Furthermore, this algorithm can better handle noise and incomplete observational data by introducing sparsity constraints as priors. However, the CS Bayesian algorithm has high computational complexity, requiring a significant amount of numerical calculations and optimization processes.

## 4. Photoacoustic Imaging Based on Compressed Sensing

The following content is the core of this paper, mainly introducing the relevant applications of CS in PAI. As mentioned above, PAI has always faced issues such as high sampling rates, limited imaging viewing angles in some cases, difficulty achieving fast real-time imaging, and high hardware costs [25,26,98,99,100]. Fortunately, CS can overcome these problems.

The limited viewing angle problem [101,102,103]: In practical applications, PAI often cannot achieve 360-degree coverage and may have a limited viewing angle. This can result in partial information being unavailable, affecting imaging quality and accuracy. CS can utilize the sparsity or structural information of signals to partially sample and effectively reconstruct signals, thereby compensating for the information loss caused by the limited viewing angle and improving imaging quality. CS can address the decrease in image quality in PAI systems due to the limited viewing angle [104,105]. 

The fast real-time imaging problem [106]: PAI systems need to acquire and process a large amount of data in a short period. Traditional methods may be time-consuming and not suitable for real-time imaging requirements. Introducing CS can accelerate signal processing speed by designing efficient sampling schemes and real-time reconstruction algorithms, thus achieving fast imaging to meet the demands of fast real-time imaging [107,108,109]. 

The hardware cost problem [58,110]: Introducing CS can save resources such as acquisition equipment and storage space [111,112]. Since CS can acquire signal information with fewer sampling points, the demand for hardware devices is reduced. At the same time, reducing data volume also saves storage space, lowers the operating costs of PAI systems, and enhances the scalability and cost-effectiveness of PAI systems. 

The image quality and accuracy problem [113,114]: CS can utilize the characteristics and prior information of signals in the reconstruction process to restore high-quality signals through optimized algorithms. This can enhance the imaging quality and accuracy of PAI, making the imaging results more reliable. Algorithms that incorporate the imaging model matrix into CS equations have special robustness [115,116].

The application of CS in PAI can be categorized into four groups. The most common method used by researchers is constructing the photoacoustic forward modeling matrix and CS equations, referred to as the physical transmission model-based CS method. Reconstruction methods using the physical transmission model have higher image reconstruction accuracy, but the computational time is relatively long. Another method involves the joint application of CS and PAI reconstruction through the CS compression measurement process, followed by conventional PAI reconstruction methods, resulting in fast imaging effects but potentially inaccurate imaging quality, known as the two-stage reconstruction-based compressed sensing method. Some researchers developed PAI algorithms from the perspective of CS principles, designed experimental devices based on single-pixel camera imaging, and conducted multiple measurements of photoacoustic signals, reducing the hardware complexity of CS-based PAI systems, referred to as the single-pixel camera-based compressed sensing method. These are the mainstream methods, but there are still valuable research approaches not classified into these categories. Based on the brief description of CS-based PAI applications provided above, we categorize all CS-based PAI methods into four types: physical transmission model-based compressed sensing method, two-stage reconstruction-based compressed sensing method, single-pixel camera-based compressed sensing method, and other valuable methods. We will provide detailed descriptions from methodological and innovative perspectives and analyze the advantages and disadvantages of each method.

**Figure 3:**
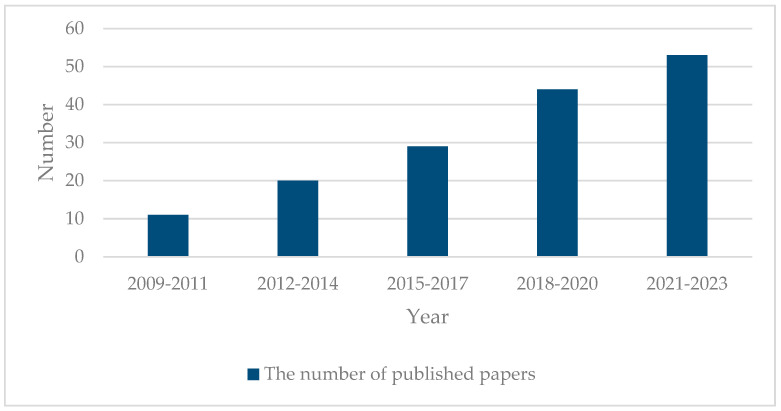
Trend of increasing publications about CS-PAI (data from Web of Science).

CS-PAI has made significant breakthroughs in the past 15 years. We have learned from the Web of Science that an increasing number of research outcomes in CS-PAI are emerging, and the quantity of articles in this field is rising year by year, as shown in Figure 3.

### 4.1. Physical Transmission Model-Based Compressed Sensing Method

As PAI algorithms research continues to advance, many time-domain and frequency-domain algorithms have emerged, such as Back Projection (BP) [35], Time Reversal (TR) [117], and Phase Shift Migration (PSM) [51,52]. The PAI algorithm based on the photoacoustic forward model is also a traditional algorithm in the photoacoustic field [118,119,120,121,122,123]. It is a model-based inversion algorithm used for two-dimensional and three-dimensional PAI. This algorithm provides an accurate and efficient forward model matrix, incorporating the imaging process into the model matrix [124,125]. This forward model matrix, also known as the physical transmission model, is used to invert and obtain images of the initial distribution of optical absorption, $P=MP_{0}$ (P is the original photoacoustic signal, M is the physical transmission model matrix, and $P_{0}$ is the initial distribution image of optical absorption). Model-based reconstruction correctly captures the effects of light attenuation through an object, suppressing artifacts (negative absorption values) that may appear in the Filtered Back Projection (FBP) algorithm.

The emergence of the physical transmission model matrix provides the possibility of integration with CS, as the physical transmission model matrix can become a part of the CS sensing matrix. The CS algorithm can transform high-dimensional original signals into related low-dimensional signals and then recover the original signals from the low-dimensional signals, and the main reason for this is that the mapping relationship of the sensing matrix connects the two. CS-PAI algorithms based on the physical transmission model mainly incorporate the forward propagation process of PAI into the sensing matrix, forming the CS equation. In 2009, Provost first introduced the concept of CS into the field of PAI [115] by directly sampling the sparsely represented initial distribution image of optical absorption and then using reconstruction algorithms for recovery. This approach reduced the number of measurements required for reconstruction. A frequency-domain photoacoustic forward model K was proposed [115]:

(8) $$K_{\left(m,n)(i,j\right)}=-ick_{n}\frac{e^{ikn\left|r_{m}-r_{ij}\right|}}{\left|r_{m}-r_{ij}\right|}g_{n}$$

where *i* and *j* are Cartesian coordinates, m is the number of transducers, *n* is the frequency, c is the speed of sound, and $r_{m}$ is the position of the transducer. K is the operator for random sampling of the Fourier domain. K is the physical transmission model. When ψ is the sparse transform basis, Kψ forms the CS sensing matrix, enabling accurate recovery of the initial distribution of optical absorption using only a small number of tomographic angles. Provost utilized various sparse base functions for CS sparse representation, comparing the performance of Fourier, numerical derivative, wavelet, and curvelet base functions in CS reconstruction. It was demonstrated that wavelet and curvelet base functions offer better reconstruction results compared to other base functions [115].

Subsequently, Wang also proposed a similar theory [116,126]. They represented the photoacoustic forward problem as y=Kx, where the matrix K is the projection matrix of PAI system, which is the physical transmission model. Similarly, the inverse problem can be written as $x=K^{-1}y$, where $K^{-1}$ represents the inverse process, and x is the initial acoustic source image. In the process of CS measurement, the measurement is incomplete. Thus, the matrix $K^{-1}$ is ill-conditioned, and $K^{-1}$ is not an exact inversion of K. In the case of insufficient measurements in PAT reconstruction, methods like back projection typically result in streak artifacts. Here, CS reconstruction methods are used to solve underdetermined systems of equations, reconstructing images with fewer measurements, which can effectively speed up data acquisition, reduce system costs, diminish streak artifacts, and enhance image accuracy. The CS-based PAT algorithm was implemented using the nonlinear conjugate gradient descent algorithm.

Building upon the photoacoustic forward model in the frequency domain, Meng proposed the photoacoustic forward model K in the time domain as the time-domain measurement matrix in CS [127]. Here, *i* and *j* are Cartesian coordinates, *m* is the number of transducers, *c* is the speed of sound, and $r_{m}$ is the position of the transducers. The time-domain photoacoustic forward model K is proposed as follows [127]:

(9) $$K_{\left(m,n)(i,j\right)}=\frac{1}{2\pi c}\delta (t-\frac{\left|r_{m}-r_{ij}\right|}{c})$$

**Figure 4:**
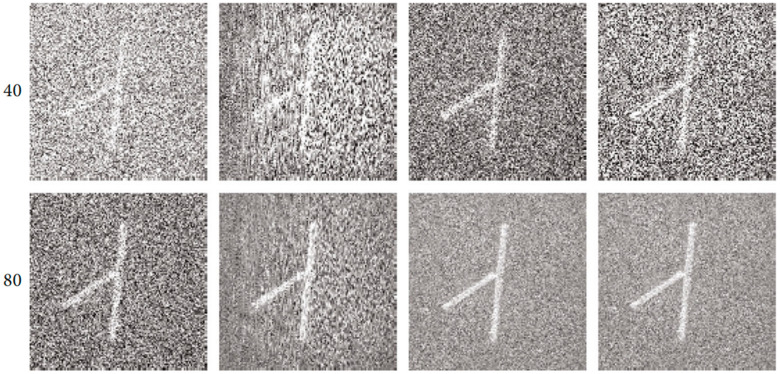
The results of agar phantom experiment. The first and second rows are the reconstruction images of carbon absorption samples from 40-view and 80-view experimental data. The first to fourth columns were reconstructed using different CS reconstruction parameters. Reprinted with permission from [128].

Since then, there have been two types of physical transmission model matrices: the time domain and the frequency domain. Both of these model matrices can reconstruct high-quality images and reduce the sampling rate of PAI systems. The main purpose is to reconstruct low-interference images with a small amount of signals. As shown in Figure 4, using a small number of channels of photoacoustic signals, the agar phantom experimental results are obtained through reconstruction using the time-domain physical transmission model [128]. 

**Figure 5:**
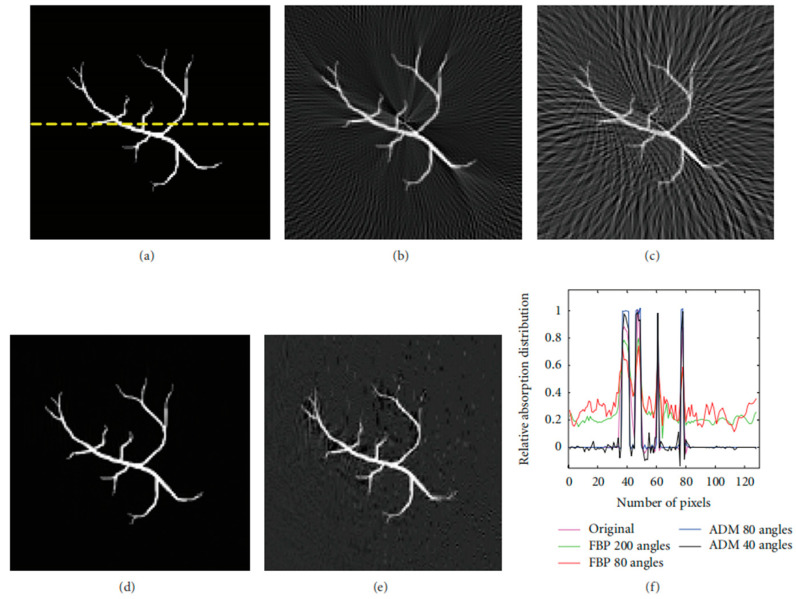
Image reconstructions using the FBP method and CS algorithm. (**a**) Original phantom. (**b**,**c**) Image reconstructions using the FBP method, with 200 and 80 transducers evenly covering the circle. (**d**,**e**) Image reconstructions using the ADM method, with 80 and 40 transducers uniformly covering the 90-degree view. (**f**) Center lines extracted from (**a**–**e**). Reprinted with permission from [109].

As shown in Figure 5, the simulated results were obtained through the physical transmission model in the frequency domain for reconstruction. At each detection angle, 64 Fourier samples were randomly selected for reconstruction within the [0.2, 2.5] MHz window. Compared with the FBP algorithm and the CS method based on the physical transmission model, the results of the CS method are significantly better than the FBP method. Furthermore, using the compressed sensing method for photoacoustic reconstruction can suppress the stripe artifacts caused by undersampling [109].

The CS sensing matrix is composed of the physical transmission model and the sparse transform basis. The physical transmission model and the sparse transform basis are incoherent, satisfying the RIP in CS. Both the time-domain model matrix and the frequency-domain model matrix are part of the CS sensing matrix, showing good performance in PAI reconstruction. In particular, frequency-domain CS reconstruction can apply low-pass filtering to PA signals, enhancing image quality and reconstruction speed, making it a promising direction for improving the quality of PAI images.

In the research mentioned above, the proposed photoacoustic forward model (physical transmission model) is an important part of the photoacoustic initial absorption distribution map obtained by inverting the CS equations. Due to incomplete measurement of the photoacoustic signals, the reconstruction process is transformed into solving a convex optimization-constrained problem. In Equation (10), X=ψS is the initial distribution of light absorption, K is the forward modeling matrix, ψ is the sparse transform basis, S is the sparse coefficient, y is the measurement value of CS, and α is the parameter controlling the reconstruction accuracy:

(10) $$\underset{x}{\min}{\left\|S\right\|}_{1},s.t.{\left\|y-K\psi S\right\|}_{2}<\alpha$$

According to Equation (10), to address the problem of recovering y to S, an appropriate reconstruction algorithm needs to be employed. In order to reconstruct high-accuracy photoacoustic images, there are many representative methods in the CS-PAI reconstruction process. For example, the fast-alternating direction algorithm is used to recover images from sparse data and noisy observation values [109]. Compared with classical methods, this method performs well in terms of computational efficiency and data fidelity, and it has a faster calculation speed. The reconstruction of S can also take advantage of the $L_{1}$ norm by introducing sparsity constraints that minimize energy deposition in the $L_{1}$ norm [129]. With this constraint in place, using convex optimization-based nonlinear recovery algorithms can significantly suppress the impact of grating lobes on imaging in the reconstruction process, effectively reducing the grating lobes generated by using linear arrays. In addition, Francis et al. exploited the signal correlation within and between signals exhibited by transducer configurations to reconstruct photoacoustic images using a distributed compressive sensing framework [130], which can achieve better image quality than model-based algorithms and reduce the number of signal samples processed. In addition to the $L_{1}$ norm, CS reconstruction based on the physical transmission model can also use the $L_{0}$ norm [131]. Moein et al. proposed a smoothed version of the $L_{0}$ norm as a reconstruction method, which can improve the peak signal-to-noise ratio of images. In the case of incomplete data, recovering the initial pressure distribution of photoacoustic imaging is often an inaccurate problem. By utilizing CS theory and sparse prior information of photoacoustic images with $L_{2}$ norm-optimization technology, combined with augmented Lagrangian weighting by alternating direction multiplier method and total variation minimization [132], integrating the sparse prior information of images into the reconstruction process effectively eliminates artifacts. It is worth noting that adding regularization terms can improve the reconstruction accuracy of solving convex optimization-constrained problems. In response to the problem of traditional regularization methods being either too smooth or too sparse, Liu et al. proposed the elastic net regularization method [128], which can improve the anti-noise ability. In specific cases, such as encountering limitations in the field of view, the split Bregman total variation algorithm can be used based on the distribution positions of all sensors [104]. This algorithm demonstrates that among all sensor arrangements, the convex sensor array performs the best, and the computation time required is also reduced. In efforts to accelerate the computation speed of model-based algorithms, researchers have used a small number of non-zero signal positions in sparse coding feature mappings as partially known supports to reconstruct photoacoustic images. This approach can maintain image fidelity in CS reconstruction while improving computational speed [133]. In the work of improving the edge resolution of photoacoustic images, researchers have developed an advanced CS reconstruction framework based on a Gaussian scale mixture model [105]. This method incorporates the structural dependencies of wavelet domain signals into the imaging framework through a Gaussian-scale mixture model. It filters the reconstructed artifacts using estimated operators, resulting in photoacoustic images with a higher signal-to-noise ratio and edge resolution. This paragraph mainly focuses on the application research of Equation (10) in the reconstruction process, aiming to improve the quality of photoacoustic images, accelerate computation speed, and reduce hardware costs. Recently, some researchers have evaluated the advantages and disadvantages of different CS-PAI reconstruction algorithms based on numerical indicators such as iteration times, CPU computing time, and SNR [109]. Table 1 compares the numerical results of the alternating direction method (ADM), L1 magic, and SPGL1 reconstruction methods, indicating that the ADM algorithm not only has a faster computation speed than the traditional L1 norm-reconstruction method but also maintains better SNR.

**Figure 6:**
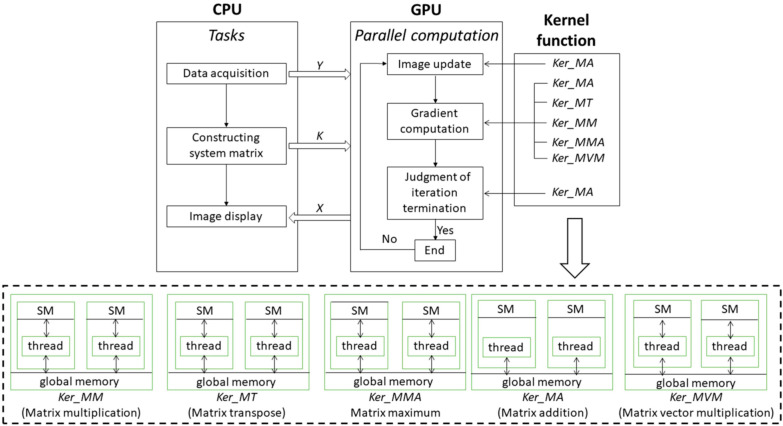
Parallel computation architecture of CS-PAT. Reprinted with permission from [107]; © Optical Society of America.

Although the CS-PAI method based on physical transmission models has many advantages, it has an undeniable limitation. The issue lies in the fact that the physical transmission model matrix K is too large. K is often a large matrix with tens of thousands of dimensions. If matrix compression is not performed, the physical transmission model matrix will consume a large amount of operating memory. In order to apply model-based CS-PAI to clinical situations, matrix-compression methods can be considered to reduce memory requirements. K is a sparse matrix, with over 99% of its entries being zeros [134]. By using the compressed sparse row format, the required memory can be greatly reduced, where nonzero entries are stored in contiguous memory locations, and the corresponding column indices are stored in an integer array. Another integer array can be used to store the index of the first nonzero entry in each row. The logic behind compressing the model matrix K is as follows: it is typically assumed that the ultrasound transducer has uniform sensitivity to detect pressure waves from 0 to 90 degrees (it typically refers to the angular sensitivity of the transducer in detecting pressure waves within a range of angles from 0 to 90 degrees with respect to the normal direction of the transducer surface). However, in reality, due to the limited size of the transducer, its sensitivity is not uniform. This results in limited detection angles, waveform distortion, and delay errors during reconstruction. To address this issue, a threshold can be set based on the incident angle, and values exceeding the threshold angle can be set to zero, thereby compressing the matrix size. By employing this approach, storage space can be reduced, and image quality can be improved. In addition to using compressed matrices to solve the problem of slow computation caused by the large photoacoustic forward model matrix K, parallel computing can also be considered to accelerate the computation speed. This method requires utilizing GPU for computation [107]. The parallel architecture process proposed in Figure 6 involves tasks assigned to the CPU, including data acquisition, matrix K construction, and display of photoacoustic images. During the reconstruction computation process, both matrix K and image data are copied from the CPU to the global memory of the GPU. The five main calculations in each iteration loop include matrix multiplication, matrix transposition, maximum value calculation, matrix addition, and matrix–vector multiplication. Among these calculations, the data required for matrix addition are directly read from the GPU’s global memory, while the data needed for the other four operations are stored in shared memory and shared among all threads within a block. Once the iterations are complete, the reconstructed photoacoustic image is transferred back from the GPU’s global memory to the CPU for image display. This achieves an image reconstruction speed 24–31 times faster than that of the CPU performance.

**Figure 7:**
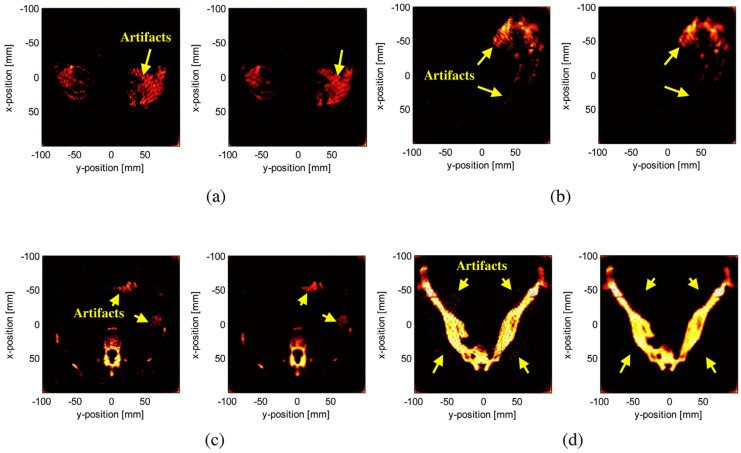
Simulation of image reconstruction with original PAT under uniform sensors (left image) and improved PAT with CS under non-uniform sensors (right image). (**a**) Breast cancer, (**b**) stomach, (**c**) intestine, (**d**) hip bone. Reprinted with permission from [136].

In the CS-PAI work based on physical transmission models, there are also some valuable applications. For example, Lin et al. proposed a model-based CS reconstruction algorithm for synthetic aperture PAI configuration [135], which enhances image resolution by enlarging the detection range through synthetic aperture methods and addresses the limited viewing angle issue. By using fewer channel signals, they can reconstruct photoacoustic images faster and achieve image quality close to that of a full-view image. To further leverage the advantages of CS reconstruction of sparse signals, Cao et al. considered using a non-uniform arrangement of annular ultrasound transducers during the acquisition of photoacoustic signals [136]. They densely distributed ultrasound transducers in the region of interest (ROI) and sparsely distributed them in the non-ROI area. This study may be helpful for clinical medical imaging applications, such as early breast cancer detection, endoscopic imaging, and in vivo monitoring. The CS-PAI technique, employing a physical transmission model, has displayed remarkable imaging outcomes for various human tissues such as breasts, stomach, intestine, and hip bones. As shown in Figure 7, the quality of the reconstructed image has significantly improved in the ROI region. The contrast regions in the four groups of images are marked with yellow arrows. Generally, the reconstructed images of traditional PAT are recognizable but have many artifacts within the ROI. In particular, numerous stripe artifacts appear around the hip bone in Figure 7d. By contrast, the image reconstructed using the method proposed in this study shows improved image quality and visual effects compared to the control image, as artifacts disappear in the same region.

Table 2 compares the traditional PAT method with the proposed CS-PAT method using three types of numerical indicators: Signal Difference to Noise Ratio (SDNR), Quality Index (Q), and Mean Square Error (MSE). The numerical results indicate that almost all indicators (highlighted in bold) show improvement when using the CS-PAT method based on the physical transmission model compared to the traditional PAT method, with the maximum percentage increase being 18.66% (the last row of Table 2). With the proposed method, it is possible to construct images of the same quality using fewer transducers, which provides the advantages of saving hardware costs and executing more efficient photoacoustic imaging for medical applications.

It is worth noting that PAI is similar to thermoacoustic imaging in principle. Algorithms based on physical transmission models are also applicable to thermoacoustic imaging. In model-based CS thermoacoustic imaging [137,138,139,140,141], a crucial step involves generating an overcomplete dictionary of the original signals to decompose the original signals into the overcomplete dictionary and the source image. Subsequently, sparse measurements are conducted on the spatial domain signals. In this method, the overcomplete dictionary serves as the physical transmission model, with the key difference being that in the CS process, the overcomplete dictionary represents an exact solution, while the physical transmission model is ill-conditioned. This approach provides new insights for CS-PAI based on physical transmission models.

Based on the CS-PAI methods utilizing the physical transmission model, the most significant advantage is the high quality and precision of the reconstructed photoacoustic images, with minimal interference from artifacts. However, these methods also have notable drawbacks, including difficulties in achieving fast real-time imaging. In the following discussion, the two-stage reconstruction-based compressed sensing method has a faster computational speed, showing the potential to achieve fast real-time imaging.

### 4.2. Two-Stage Reconstruction-Based Compressed Sensing Method

The physical transmission model-based compressed sensing method can reconstruct the image from the measurement data directly, but it also takes more time and has to modify the sampling strategy [142]. The CS methods combined with standard reconstruction methods, also called two-stage reconstruction-based compressed sensing methods, have higher speeds and lower complexity. In the first step the point-wise pressure has been recovered from the CS measurements. In the second step, a standard reconstruction method, such as BP or TR, has been used to obtain the photoacoustic source [143]. 

Two-stage reconstruction methods typically focused on the first step. Haltmeier et al. used expander matrices as the measurement matrices to satisfy their random nature [108,144,145]. Besides this, they also found a sparsifying temporal transform in the case of circular geometry to ensure the sparsity of the signal. It can be described as [108]

(11) $$T(p):=t^{3}\partial_{t}t^{-1}\partial_{t}p$$

Considering that the transform T only acts in the temporal variable while the measurement matrix A acts in the spatial variable, T and A can be exchanged:

(12) T(y)=A(Tp)

**Figure 8:**
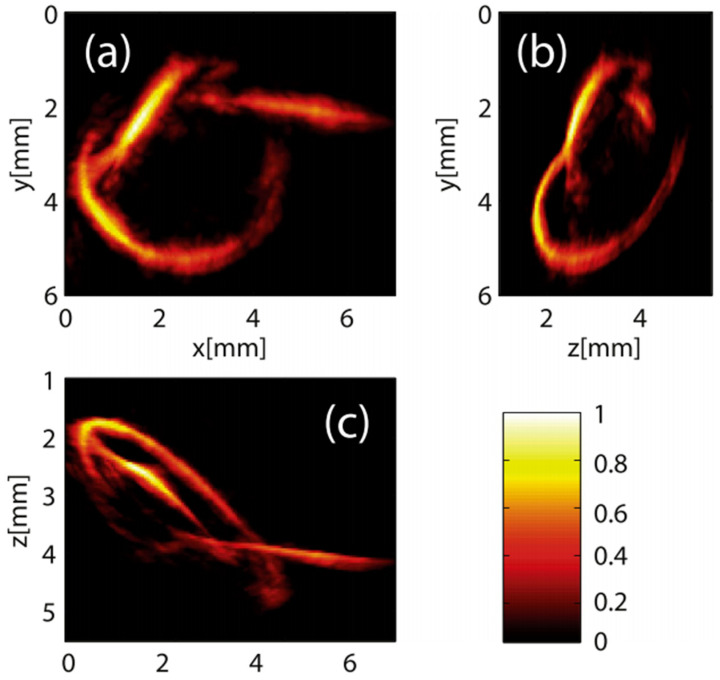
Reconstruction results utilizing compressed sensing measurements. Maximum intensity projections of a silicone loop in the z-direction (**a**), x-direction (**b**), and y-direction (**c**). Reprinted with permission from [108].

The proposed CS scheme’s effectiveness is validated through reconstruction results from experimental data, as shown in Figure 8. These results demonstrate that the method can indeed reduce the number of spatial measurements required without sacrificing spatial resolution, thereby potentially increasing the imaging speed in PAI.

Similar to the study of Haltmeier et al., P. Burgholzer et al. also proposed a sparsifying temporal transform [145]:

(13) $$T^{\prime }=\partial_{r}rH_{r}\partial_{r}$$

where $H_{r}$ represents Hilbert transform. The transform $T^{\prime }$ is applied to the spherical means M. $M\left[f\right]$ and the measurement photoacoustic signal p(t) are equivalent when used to recover the initial pressure [146]. 

**Figure 9:**
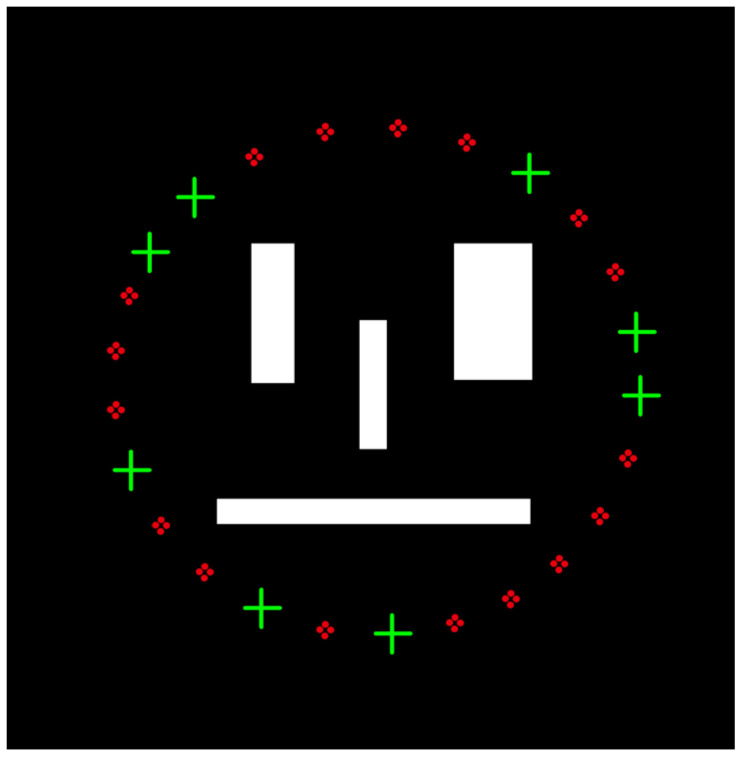
Measurement signals are random zero (green ‘+’)/one (red dot) combinations of individual line detectors.

Further, they used a 64-channel detector array with fiber optic line detectors and 16 fiber-optical Mach–Zehnder interferometers. The measurement chamber contains 64 fiber optic line detectors, with 16 channels read out in parallel using 4 × 1 switches, and the measurement signals are random zero/one combinations of individual line detectors, as shown in Figure 9. This setup allows for the acquisition of high-quality images while reducing the acquisition time and system costs through compressed sensing techniques. Overall, they presented a new perspective on photoacoustic imaging by incorporating compressed sensing principles and a sparsifying transform. This offers the advantages of potentially reducing measurement requirements, improving recovery guarantees, and enhancing image reconstruction quality, as supported by theoretical analysis and simulation results.

Still focusing on the issue of signal sparsity, Zhou et al. utilized a transform matrix obtained through the learning of a dictionary with the K-SVD method [147,148]. As shown in Table 3 and Table 4 [147], visual assessment and quantitative evaluations, including metrics such as MSE and peak signal-to-noise ratio (PSNR), demonstrate the superiority of the proposed method. They offered a novel and promising approach to address the challenges in PAI, leveraging compressed sensing principles and a learned dictionary to enhance image reconstruction quality and overcome limitations imposed by hardware constraints.

Endoscopic photoacoustic tomography is an interventional application of photoacoustic tomography designed to visualize anatomical features and functional components within biological cavity structures, such as the nasal cavity, digestive tract, or coronary arterial vessels. It also has the challenge that the acoustic measurements are incomplete due to limited detectors or restricted acoustic detection views within the cavity. This limitation often leads to degraded image quality when using conventional image reconstruction methods. Also, through the learning of a dictionary, Sun et al. [149] applied the two-stage method to PAE for reconstructing high-quality images that depict the initial pressure distribution on a cross-section of the cavity from incomplete discrete acoustic measurements. This method involves constructing and training a comprehensive dictionary for the sparse representation of the acoustic measurements induced by photoacoustic signals. By optimizing the sparse measurements and a sensing matrix, the sparse representation of the complete acoustic signals is obtained, followed by the recovery of the complete signals through inverse sparse transformation. The image of the initial pressure distribution is then reconstructed using the time reversal algorithm based on the recovered complete signals. Their numerical experiments demonstrate that high-quality images with reduced under-sampling artifacts can be reconstructed from sparse measurements using the proposed method. Comparative results indicate that the proposed approach outperforms standard TR reconstruction by 40% in terms of the structural similarity of the reconstructed images.

It is worth mentioning that Marta et al. proposed a new two-stage method [150]. Instead of point-by-point recording, a single-pixel optical camera has been used so that the entire active area of the entire active area of the optical ultrasound sensor is illuminated. They recovered the photoacoustic data at each time step independently from patterned measurements. This recovery process leverages the sparsity of the data when represented on a Curvelet basis [151], which is particularly effective for capturing the inherent geometric features of photoacoustic signals. Following the successful recovery of these sparse representations, the next step involves reconstructing the PAI using standard PAT reconstruction methods. This approach allowed for a more efficient handling of the data, potentially leading to enhanced image quality and reduced computational demands by initially focusing on the sparsity-constrained recovery of the signal before proceeding with conventional PAT image-reconstruction techniques.

Also inspired by the principle of single-pixel cameras, Arridge et al. explored two general spatial sub-sampling strategies, donated by rSP-Msub and sHd-Msub, and their implementation using the Fabry–Pérot (FP) interferometer [143]. The incident photoacoustic field on the detection plane, p(x=0,y,z,t), caused by the *j*th pulse of the excitation laser, is first multiplied by a spatial window function ϕ(y,z) and then integrated over the whole detection surface:

(14) $$f_{i}(t)=\int p(x=0,y,z,t)\phi_{j}(y,z)dydz$$

The spatial sampling is followed by temporal sampling, e.g., by measuring $f_{i}(t)$ at $t_{i}=i_{t}\delta_{t},i_{t}=1,\ldots ,M_{t}$.

**Figure 10:**
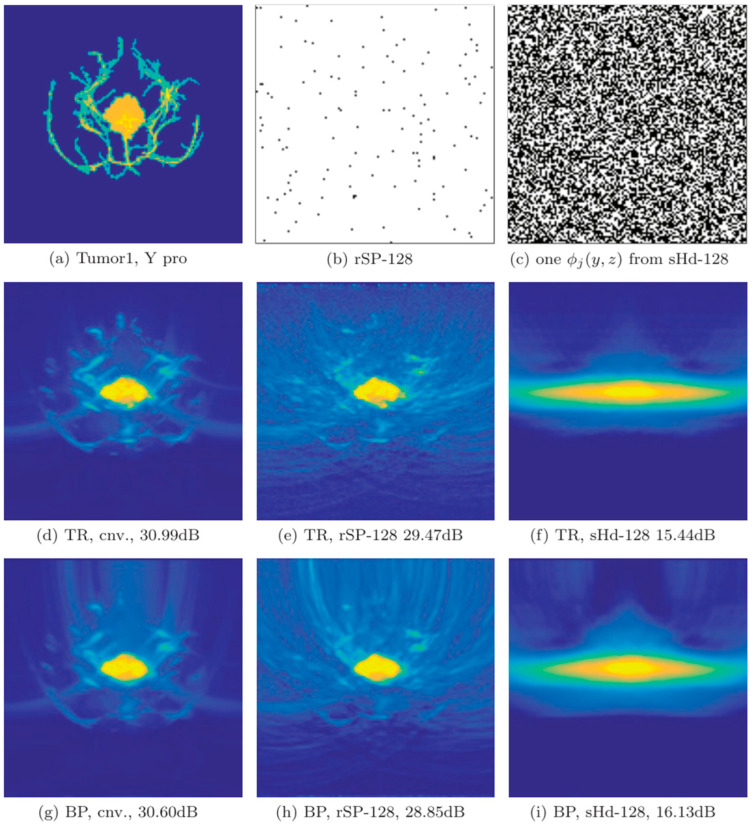
(**a**) Phantom. (**b**) Visualization of the rSP-128 sub-sampling pattern. (**c**) The sHd-128 pattern. (**d**–**i**): TR and BP results for conventional data (left column), rSP-128 (middle column), and sHd-128 (right column) and their corresponding PSNR in dB. Reprinted with permission from [143].

Their work also involves improving compressed sensing reconstruction methods. Through the utilization of compressed sensing methodologies and variational image reconstruction algorithms like total variation regularization combined with Bregman iterations, high-quality images can be reconstructed from significantly sub-sampled PAT data. These approaches not only boost acquisition speeds for point-by-point sequential scanning configurations but also decrease the number of channels needed for parallelized schemes utilizing detector arrays. The research findings demonstrate the promise of these innovative compressed sensing PAT devices through outcomes obtained from simulated data, dynamic experimental phantoms, and in vivo experiments. The results can be seen in Figure 10. This work indicates that by employing appropriate sparsity constraints and advanced reconstruction methodologies, it is viable to achieve high spatial resolution and contrast in 4D PAT, paving the way for advancements in PAI.

Despite these advantages, the two-stage methods still need a lot of detector locations to realize multiple measurements. In order to compensate for this shortcoming, single-pixel cameras have been combined with CS methods, so that only one detector is needed among the process of measurements. These will be introduced in detail in the next section.

### 4.3. Single-Pixel Camera-Based Compressed Sensing Method

**Figure 11:**
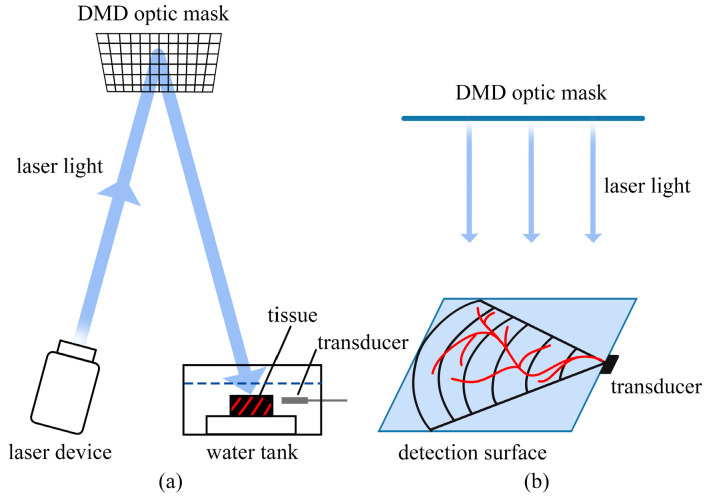
The schematic of the CS method for PAI with one ultrasonic transducer. (**a**) Compressed data acquisition. (**b**) PA signal reception.

Single-pixel cameras capture images using a single detector element. They modulate incident light with patterns and measure the resulting intensity. CS algorithms then reconstruct the original image from these measurements, exploiting sparsity in natural scenes. This approach reduces sampling requirements and enables cost-effective imaging [152,153,154]. The imaging principle of single-pixel cameras can be applied to PAI. As shown in Figure 11 [155], an optic mask is placed above the laser source and the tissue to carry out the laser-encoded emission. The tissue is irradiated by the encoded laser light and absorbs the optic energy to generate the acoustic waves. 

Dong et al. [156] utilized spatially and temporally varying random optical illumination instead of uniform illumination typically used in conventional PAI methods. CS is employed to reduce the number of random illuminations for faster data acquisition. They obtained a new forward model that can be described as [156]

(15) $$\tilde{p}(r_{0},k,m)=-ik\iint_{s^{\prime }}dS^{\prime }G_{k}^{out}(r^{\prime },r_{0})I_{m}(r^{\prime })p_{0}(r^{\prime })$$

where $I_{m}(r^{\prime })$ is the random illumination on the object at location $r^{\prime }$ from the *m*th mask. The images can be reconstructed from acquisitions with as few as two transducer view angles. The proposed acquisition and reconstruction scheme offers several key advantages, including improved image quality, faster data acquisition, reduced hardware requirements, cost-effectiveness, and innovation in the approach to illumination. These advantages establish the scheme as a promising advancement in the field of limited-view PAI with the potential for widespread impact on imaging quality and system efficiency.

Huynh et al. used a single-pixel camera for three-dimensional compressed-sensing photoacoustic tomography. This is achieved by reflecting a large collimated laser beam from a planar Fabry–Pérot ultrasound sensor onto a digital micromirror device, which then patterns the light using a scrambled Hadamard basis before it is collected into a single photodetector. The inner products of the Hadamard patterns and the distribution of thickness changes of the ultrasound sensor induced by photoacoustic waves are recorded. An accelerated proximal gradient-type algorithm with total variation regularization is used to directly recover the initial distribution of acoustic pressure generating those waves from the measured signals. This approach demonstrates the ability to obtain three-dimensional PAT of imaging phantoms with compression rates as low as 10%, showcasing the potential of compressed sensing techniques to reduce data acquisition time and volume, which is crucial for developing faster imaging systems with higher resolution and larger fields of view [157].

Contrary to the work of Huynh et al., Wu et al. used edge-expander codes-based masks rather than ordinary random masks to increase the number of the measurements of the important elements and efficient total variation regularization-based model to improve the capability of CS-PAT for reducing the number of measurements and fast data acquisition. The proposed method is shown to have better reconstruction quality and significantly lower computing times compared to other methods [158]. 

Despite the attainment of certain outcomes, hurdles persist in single-pixel PAI, particularly when aiming for high spatial resolution approaching the deep-subwavelength regime of acoustics. Guo et al. proposed a dual-compressed PAI technique [111]. In addition to spatially patterned illumination, this approach uses a coded acoustic aperture to distinguish temporally the time-of-flights (TOF) of spatial-dependent photoacoustic signals. According to the Poisson–Kirchhoff principle [159], the detected photoacoustic signal u(t) is a linear superposition of photoacoustic waves $C_{ij}(t)$ originating from different spatial positions (i,j) in the tomographic image of a 3D object [160]. The aperture introduces a time delay $T_{ij}$, which yields the modulated signal $C_{ij}^{\prime }(t^{\prime })$ and forms the recorded superposed signal $u_{ij}^{\prime }(t^{\prime })$. The new TOF $t^{\prime }=t+T_{ij}=g_{ij}t$.

The amplification factor:

(16) $$g=\langle g_{ij}\rangle =\langle 1+T_{ij}/t\rangle \approx (g_{\max}+1)/2$$

The CS process can be described as

**Figure 12:**
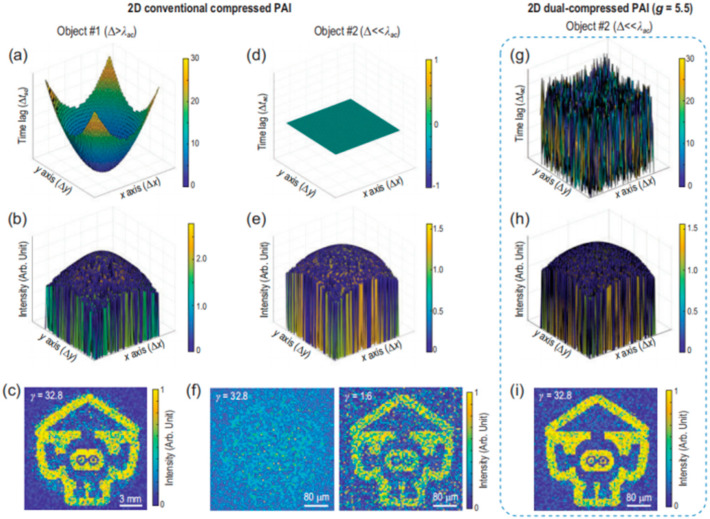
(**a**,**d**,**g**) Time-lag distribution, (**b**,**e**,**h**) intensity distribution, and (**c**) the recovered image acquired from imaging of Object #1 (pixel size Δ = 200 μm > $\lambda_{ac}$, and $\lambda_{ac}$ is the wavelength of the acoustic detection in water) with a compression ratio of γ = 32.8. (**f**) Object #2 (Δ = 5 μm << $\lambda_{ac}$), with γ = 32.8 and γ = 1.6, (**g**–**i**) dual-compressed, single-pixel PAI. (**i**) Recovered image acquired from imaging of Object #2, with γ = 32.8. Reprinted with permission from [111].

(17) u=Ty+n=TSv+n=Hv+n

where *u* represents the detected signal, and v is the image after basis sparse representation. The matrices S and T are produced by optical sensing and acoustic sensing, and n is the noise. As is shown in Figure 12 [111], when it comes to a smaller pixel size (much smaller than the wavelength of the acoustic detection in water), this method can also produce images of high quality and compression ratio. The dual-compressed photoacoustic imaging with single-pixel detection offers advantages in terms of efficiency, spatial resolution, innovative transformation of spatial differences, generalizability to other imaging modalities, and empirical demonstration of its capabilities. These advantages position the approach as a promising innovation in the field of PAI, with potential implications for a wide range of imaging applications.

**Figure 13:**
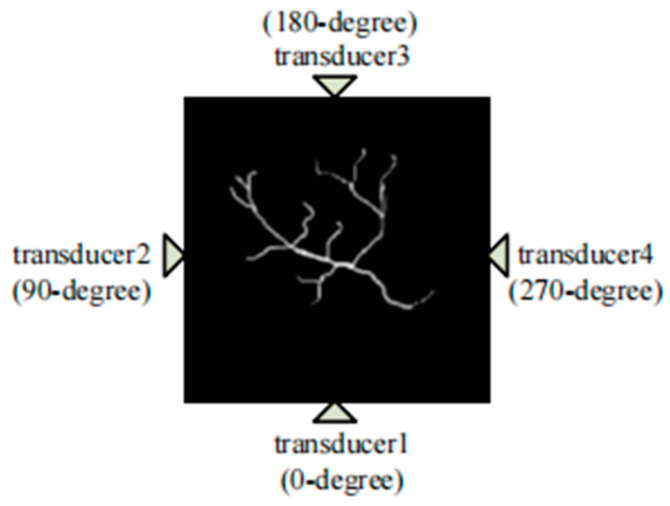
Transducers’ different observation locations using CS method for PAI based on multiangle observation. Reprinted with permission from [155]; © Optical Society of America.

On the basis of traditional single-pixel camera PAI methods, Sun et al. further investigated a new approach that combines the arc direction and random optical illumination [155]. In their work, they first employed random illumination patterns and then considered the issue of the receiver angle in the detection process. To solve the problem of visible artifacts and loss of details, four single-element transducers were adopted and placed at different degrees (0, 90, 180, and 270 degrees), which is shown in Figure 13. For each detection unit, only the ultrasound signals within the detection angle could be detected. 

**Figure 14:**
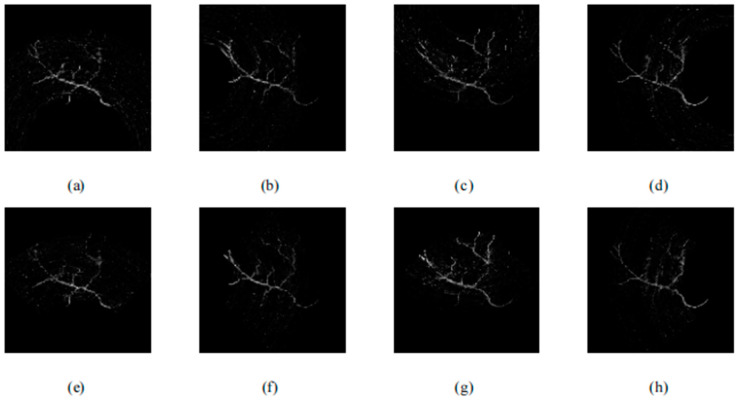
Imaging results reconstructed by rectangle CS methods. (**a**–**d**) 0-degree, 90-degree, 180-degree, and 270-degree adoption using rectangle CS method, respectively; (**e**–**h**) 0-degree, 90-degree, 180-degree, and 270-degree adoption using arc-direction CS method, respectively. Reprinted with permission from [155]; © Optical Society of America.

**Figure 15:**
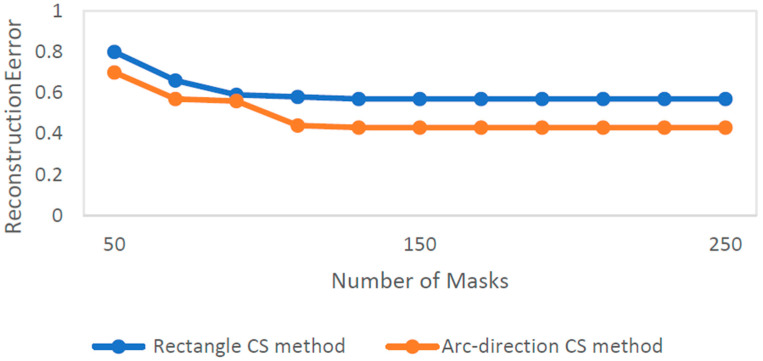
Reconstruction errors based on rectangle and arc-direction CS methods in the case of 270-degree adoption. This figure is adapted from [155].

Unlike conventional approaches that rely on rectangle CS methods, this innovative technique offers a more sophisticated way to compress PA data along the arc line, allowing for the accurate reconstruction of PA images from observations at multiple angles. The results can be seen in Figure 14 and Figure 15. By incorporating the arc-direction mask and CS reconstruction algorithm, this method aims to enhance the fidelity of reconstructed images by preserving important structural details that might otherwise be lost or distorted. Through comprehensive simulation studies, the effectiveness of this approach has been validated, showcasing its potential for advancing high-resolution PAI applications.

The mentioned arc-direction mask and arc-direction CS reconstruction algorithm in PAI provide advantages such as addressing data incompleteness artifacts, preserving image details, enhancing spatial resolution, applicability across diverse scenarios, and facilitating artifact-free imaging [155]. These advantages position the method as a valuable contribution to advancing the capabilities of PAI and improving the quality of PAI outcomes.

### 4.4. Other Valuable Methods (Virtual Detector, Multiscale Decomposition of Wave Equation, Motion Estimation Framework, Undersampled Fourier Measurements, Laplacian Sparsity, and Deep Learning)

In the previous sections, we have introduced three major categories of CS-PAI methods. However, there are still some methods that cannot be classified into these categories. Nevertheless, these unclassified methods also hold important application value and research significance [161,162]. 

For example, traditional PAM suffers rapid degradation in image quality outside the ultrasound focal region when using a focused ultrasound transducer for photoacoustic detection. In order to improve the imaging quality of PAM in the defocused region, a CS-based virtual detector photoacoustic microscope was developed. The focus of the ultrasound transducer is regarded as a virtual detector [163], and mechanical scanning of this virtual detector is performed, similar to the detection with a linear ultrasound array. This allows for the recovery of information in the defocused region, thereby enhancing the imaging quality in the defocused area.

Zangerl et al. derived a multiscale decomposition of the wave equation and applied it to CS-PAT [164]. They proposed a multiscale reconstruction method that reconstructs images from a few CS measurements consisting of linear combinations of signals recorded by a single detector. This method utilizes the principle of acoustic reciprocity to achieve the desired output sound pressure through the application of operators acting on acoustic data in the time domain for multiscale decomposition. By introducing sparsity of the desired initial photoacoustic pressure distribution at high-frequency scales in this manner, significant progress has been made.

CS can improve the problem of a long acquisition time in high-resolution 3D PAT scanners. Lucka et al. developed a joint reconstruction and motion estimation framework to further enhance the quality of dynamic PAT images [165]. They employed sparse image reconstruction with sparsity-constrained motion estimation models and utilized the temporal redundancy of data, resulting in images with good spatial resolution and contrast.

To prove the applicability of CS in PAT, known reconstruction formulas were applied in models of wave propagation in free space and bounded domains, along with theoretical frameworks of Riesz bases and non-uniform Fourier series. This simplified the inverse problem into a CS problem of undersampled generalized Fourier measurements [166]. Although the research is still in its preliminary stage, these findings pave the way for future studies. Two possible extensions were discussed, including generalizing the results to broader domains and further theoretical exploration of CS problems with subsampling patterns of specific structures. Additionally, numerical evidence regarding methods based on Riesz sequences was provided, but rigorous stability proof of this method remains a subject for future work.

**Figure 16:**
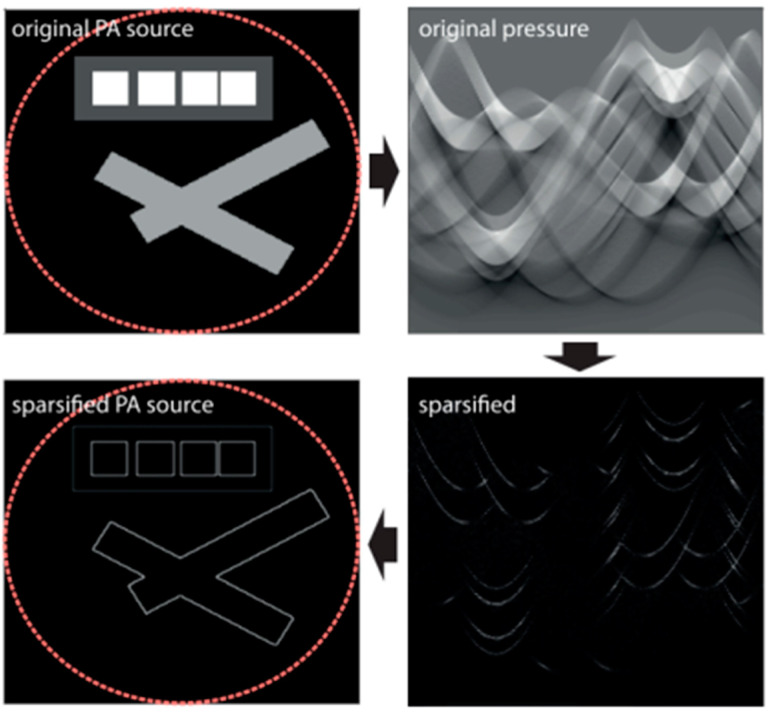
(**Top left**) The initial photoacoustic source, (**top right**) the initial photoacoustic signal, (**bottom right**) the initial photoacoustic signal after Laplacian sparring, and (**bottom left**) the edge information of the photoacoustic source reconstructed with the sparse initial photoacoustic signal. Reprinted with permission from [167].

One important role of CS is to reduce the sampling rate of PAI systems while maintaining high spatial resolution. CS does not involve point-to-point measurements but rather various combinations of pressure values from different sensor positions. Sparsity is a key condition that allows for the recovery of the photoacoustic source from compressed measurements. There are many ways to sparsify the original photoacoustic signal, and a particular sparsification method can achieve edge detection of the imaged object [167]. This method utilizes the second derivative of the measured acoustic pressure data, where the second derivative corresponds to the application of the Laplace function on the original photoacoustic source. Since conventional photoacoustic sources consist of smooth parts and singular points along interfaces, the Laplace function of the source is sparse. As shown in Figure 16, this process effectively sharpens the edges of the original photoacoustic source. 

**Figure 17:**
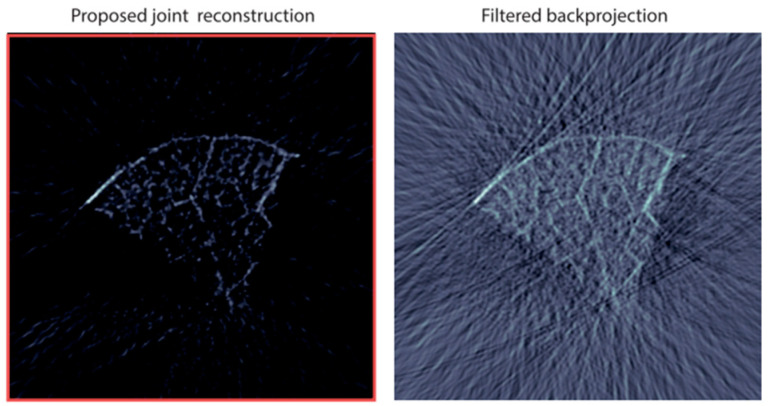
The experimental data were reconstructed using 60 sparse samples: (**left**) PAT image reconstructed using the joint method; (**right**) FBP reconstruction. Reprinted with permission from [167].

To effectively leverage sparsity, Haltmeier developed a reconstruction framework to jointly recover the initial photoacoustic source and the modified sparse source, thereby integrating edge information of the photoacoustic source with information inside the edges [167]. As shown in Figure 17, the joint reconstruction algorithm significantly reduces the interference of artifacts compared to the FBP algorithm under the same conditions.

**Figure 18:**
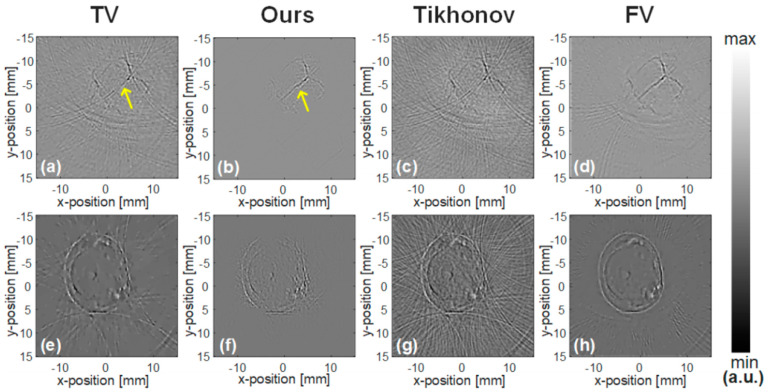
The in vivo results with 50% sub-sampling rate, (**a**–**d**) the results of mice brain, in which the yellow arrows indicate the vessels in sulcus, and (**e**–**h**) the results of cross-section of finger. (**a**,**e**) The iterative total variation method; (**b**,**f**) the mentioned approach with TV prior; (**c**,**g**) the iterative Tikhonov method; (**d**,**h**) the full-view results. FV: full-view. Reprinted with permission from [169].

With the continuous development of deep learning theory, there is great potential for the integration of deep learning and CS. Possibilities include using deep learning to construct sparse representations of signals, designing neural network models for CS reconstruction, optimizing CS sampling strategies using reinforcement learning methods, and utilizing deep learning for compressive sensing of multimodal data. Researchers have already combined deep learning with CS-PAT, such as using NETT regularization to solve CS-PAT problems [168]. These methods use forward operators at each iteration. However, there are signal differences in this process, indicating that simulated training data differ from actual measured real data. Developing more accurate forward models and improving training data are important goals for the future. Gao et al. proposed a new method for CS-PAT using untrained neural networks to suppress image artifacts or sidelobes caused by a small number of measurement signal channels or limited viewing angles in the CS process [169]. This method can reduce half of the measured channel numbers and recover sufficient details using neural networks for reconstruction. No additional learning based on deep image prior information is required. The model only needs a small amount of gradient descent detection to reconstruct images. As a non-learning strategy, this method can be combined with other existing regularization methods to further improve image quality. Validations have shown that under the same regularization conditions, as shown in Figure 18, this untrained neural network method outperforms traditional compressive sensing methods.

## 5. Comparisons between Different Methods

The single-pixel camera-based compressed sensing technique is often used in conjunction with other methods, primarily for low-cost multiple sampling rather than to impact image quality and efficiency. In the study of Bolin et al., two-stage methods and physical transmission model-based CS methods were subjected to an identical set of conditions, and their imaging results were compared in Table 5. This allowed for an analysis of the advantages and disadvantages of each method [170]. The major benefit of any two-stage approach is that it decouples the nonlinear iterative compressed sensing recovery and the linear acoustic propagation problems; thus, the expensive acoustic forward and adjoint operators are not iterated with. At the same time, the physical transmission model-based CS method performs better in terms of image quality.

**Figure 19:**
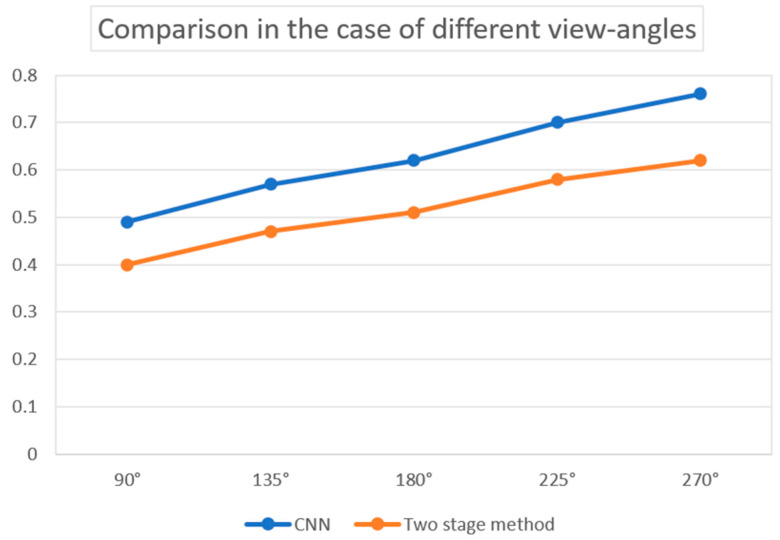
SSIM comparison for the images reconstructed with the proposed method and the CNN method in the case of different view angles. This figure is adapted from [149].

Otherwise, Sun et al. compared the two-stage method with the deep learning algorithm [149]. In their work, structural similarity (SSIM) was used to evaluate the quality of imaging. The result can be seen in Figure 19, and it demonstrates the potential of the convolutional neural network (CNN) method over the CS method in reconstructing structures even in non-detection areas. While the CS method can recover unmeasured acoustic signals in the scanned area from sparse measurements instead of signals in the non-detection area, it may lead to loss of structure information in the non-detection area. In contrast, a well-trained CNN can recover partial or complete lost information based on prior knowledge of imaging structures, showing significant potential in reconstruction.

## 6. Discussion (Challenges and Future Perspectives)

CS has been applied in PAI for over a decade, yielding significant achievements. Numerous results have demonstrated that CS plays a crucial role in reducing the sampling scale of PAI systems, enhancing the acquisition speed of photoacoustic signals, lowering hardware costs, improving the accuracy of images (especially in limited viewing angle scenarios), and mitigating the impact of artifacts on images. The development of CS is significant for expanding the clinical applications of PAI, especially in fields with high demands for imaging speed, such as hemodynamics, oxygen metabolism, physiological monitoring, and in vivo drug detection. Fast PAI systems are needed for real-time tracking and collecting information reflecting the status of biological tissues. By utilizing CS technology, it is possible to decrease the amount of signal sampling and acquisition time, thereby achieving rapid imaging and providing more efficient and real-time imaging solutions for related clinical research. Additionally, in clinical applications for disease diagnosis, improving the data acquisition speed is also crucial. Reducing the time patients spend using imaging devices can minimize exposure to microwave radiation, enabling timely acquisition of disease information and targeted treatment. This significantly impacts the accuracy and efficiency of clinical diagnosis and treatment.

However, CS-PAI also faces some challenges. Although the introduction of CS has improved the speed of signal acquisition, it requires solving a nonlinear objective function. The solution to the objective function is an iterative process that may require dozens or even hundreds of iterations to achieve convergence of the image, especially when using algorithms based on physical transport models. When the dimensionality of the photoacoustic signal is large, the reconstruction speed becomes very slow. To address this issue, one can consider using high-performance computers combined with GPU acceleration to significantly improve the speed of the reconstruction process.

Furthermore, the compression measurement process is difficult to implement in hardware. When designing an optimized algorithm for CS-PAI, the ultimate goal is to make the algorithm hardware-based and turn it into a mass-produced product. However, the information content in the sensing matrix generated during the CS compression measurement process may occupy more memory than the original signal, making it difficult to transmit the sensing matrix. To address this issue, one can consider compressing the memory occupied by the sensing matrix. This has always been a key focus area in CS.

In terms of stability, CS-based PAI still suffers from limited instability and robustness currently. When reconstructing signals with small dimensions using CS, random noise is always generated. In PAI, if the imaging area is small, it is easy to generate aliasing artifacts during reconstruction. There are even cases where aliasing artifacts appear at different positions in the image each time, even when the reconstruction parameters are the same. This is because signals with small dimensions are not easy to converge to an exact solution. As the signal dimension increases, the aliasing artifacts will disappear, but the time cost will also increase. Optimizing more stable algorithms is crucial for improving the quality of reconstructed images.

In the CS equation, the sparse representation basis is an important factor influencing the reconstruction accuracy of CS. Constructing a sparse basis that can transform an unknown acoustic source vector into a sparse vector is challenging and represents an uncontrollable factor due to the lack of prior information about the acoustic source vector. As mentioned above, commonly used sparse bases include Fourier bases, wavelet bases, numerical derivatives, and discrete cosine bases. However, these sparse bases cannot guarantee to make any unknown acoustic source vector sparse. Therefore, in future work, it may be considered to design a sparse singular matrix using the pseudo-inverse matrix of this sparse singular matrix as the sparse basis aims to make any acoustic source vector sparse.

## 7. Conclusions

In the Introduction section of this article, the feasibility of CS in the field of PAI was fully demonstrated. The combination of CS and PAI can solve multiple critical biomedical issues and has achieved significant results. By utilizing sparse representation and reconstruction algorithms, CS effectively reduces the sampling rate of PAI systems and shortens the acquisition time of photoacoustic signals. The introduction of CS brings a more efficient way of data acquisition to PAI systems and even allows for selecting specific channel signals for photoacoustic reconstruction through CS sub-sampling. Concerning the limited viewing angle problem, CS can compensate for the decrease in image quality caused by a limited viewing angle by utilizing the sparsity or structural information of signals for partial sampling and reconstruction, achieving imaging effects close to full-view imaging. One of the most significant advantages of CS in PAI is meeting the demand for fast imaging. By designing efficient sensing matrices and real-time reconstruction algorithms to accelerate signal processing speed, CS meets the requirements for fast real-time imaging. In terms of hardware resource costs, CS reduces the requirements on the sampling end, thus reducing the need for hardware devices and storage space, enhancing the economy and scalability of PAI systems. During the reconstruction process, CS utilizes signal characteristics and prior information to restore high-quality signals through optimization algorithms, improving the reliability and accuracy of imaging results, thereby bringing new possibilities and advantages for the development and application of PAI systems. It is expected that more valuable work based on CS-PAI will emerge in the future.

## Figures and Tables

**Table 1:** Numerical results of L1 magic, SPGL1, and ADM methods on CS-PAI images with 16–80 transducers uniformly distributed over a 90-degree view. Reprinted with permission from [109].

Positions	Iterations			Experimental CPU Time (s)			SNR (dB)		
	Magic	SPG	ADM	Magic	SPG	ADM	Magic	SPG	ADM
16	67	340	88	471.2	16.3	5.5	−3.8	−3.6	−3.5
24	75	422	73	692.1	28.7	3.4	0.7	0.9	3.9
32	77	345	68	831.5	29.5	7.9	3.3	3.4	3.6
40	78	350	58	877.5	37.5	6.6	11.1	12.l	13.4
48	80	337	53	1280.7	43.2	9.3	11.1	11.7	13.8
56	79	317	46	1294.2	45.3	4.1	20.3	24.6	28.6
64	79	429	43	1179.6	64.5	5.0	20.4	22.1	25.9
72	81	367	46	1711.9	66.4	6.0	21.2	22.9	28.7
80	84	366	42	1580.9	68.7	11.4	22.7	29.1	29.7
Average				1102.2	44.5	6.6			

**Table 2:** Comparative reconstruction results of four types of human tissues using traditional PAT method and CS-PAT method based on physical transmission model. Reprinted with permission from [136].

		SDNR	Q	MSE
Breast cancer	Traditional PAT	0.8792	0.3070	0.1140
	Improved PAT + CS	0.9125	0.3443	0.1044
	Improvement (%)	**3.79**	**12.15**	**8.42**
Stomach	Traditional PAT	1.7540	0.4020	0.0815
	Improved PAT + CS	1.8225	0.4395	0.0739
	Improvement (%)	**3.91**	**9.33**	**9.33**
Intestine	Traditional PAT	0.6494	0.3280	0.1093
	Improved PAT + CS	0.6680	0.3785	0.1000
	Improvement (%)	**2.86**	**15.40**	**8.51**
Hip bone	Traditional PAT	0.3550	0.3511	0.0648
	Improved PAT + CS	0.3517	0.4166	0.0564
	Imp roveme nt (%)	−0.93	**18.66**	**12.96**

**Table 3:** MSEs for different reconstruction methods. This table is adapted from [147].

Points	Direct Reconstruction	CS with DCT	CS with K-SVD
150	0.003	0.002	0.002
100	0.008	0.004	0.003
50	0.017	0.012	0.001

**Table 4:** PSNRs for different reconstruction methods. This table is adapted from [147].

Points	Direct Reconstruction	CS with DCT	CS with K-SVD
150	73.085	75.313	76.039
100	69.006	72.432	73.280
50	65.804	67.208	68.224

**Table 5:** Evaluation comparison among time reversal and two CS-PAI methods. The table is adapted from [170].

	Time Reversal	Two Stage Method	Physical Transmission Model Based CS Method
MSE	0.026	0.011	0.003
SSIM	0.553	0.621	0.791
PSNR	16.023	18.703	25.005
SNR	18.817	22.867	25.272

## Data Availability

The data presented in this study are available on request from the corresponding author.
